# Affordability of adult HIV/AIDS treatment in developing countries: modelling price determinants for a better insight of the market functioning

**DOI:** 10.7448/IAS.19.1.20619

**Published:** 2016-10-19

**Authors:** Luis Sagaon-Teyssier, Sauman Singh, Boniface Dongmo-Nguimfack, Jean-Paul Moatti

**Affiliations:** 1 Aix Marseille Univ, INSERM, IRD, Sciences Economiques & Sociales de la Santé & Traitement de l'Information Médicale (SESSTIM), Marseille, France; 2 ORS PACA, Observatoire régional de la santé Provence-Alpes-Côte d'Azur, Marseille, France; 3 HIV Department, World Health Organization, Geneva, Switzerland

**Keywords:** HIV-treatment, price determinants, originator and generic medicines, patents

## Abstract

**Introduction:**

This study aims to provide a landscape of the global antiretroviral (ARV) market by analyzing the transactional data on donor-funded ARV procurement between 2003 and 2015, and the ARV price determinants.

**Design:**

The data were obtained from the Global Price Reporting Mechanism (GPRM) managed by the AIDS Medicines and Diagnostics Service of the WHO, and it consists of information that covers approximately 80% of the total donor-funded adult ARV transactions procurement.

**Methods:**

ExWorks prices and procured quantities were standardized according to the guidelines in terms of yearly doses. Descriptive statistics on quantities and prices show the main trends of the ARV market. Ordinary least squares estimation was carried out for the whole sample, then stratified according to the type of supplier (originator and generic) and controlled for time and geographical fixed-effects. Given that analyses were carried out on a public dataset on ARV transactional prices from the GPRM, ethics are respected and consent was not necessary.

**Results:**

Originator medicines are on average the least expensive in the sub-Saharan Africa region, where at the same time, generic medicines are on average the most expensive. By contrast, originator medicines are the most expensive in Europe and Central Asia, and generic medicines are the least expensive. In fact, the data suggest mixed strategies by ARV suppliers to exploit opportunities for profit maximization and to adapt to the specific conditions of market competition in each region. Our results also suggest that the expiration of patents is not sufficient to boost additional developments in generic competition (at least in the ARV market) and that formal or informal agreements between generic firms may *de facto* slow down or even reverse long-term trends towards price decreases.

**Conclusions:**

Our findings provide an improved understanding of the ARV market that can help countries strengthen policy measures to increase their bargaining power in price negotiations and the use of TRIPS flexibilities, with a special emphasis on negotiations with generic manufacturers.

## Introduction

The prices of first-line antiretroviral (ARV) medicines fell significantly in recent years, allowing for the scaling up of access to ARV treatment in the developing world. In 2012, nearly 9.7 million people living with HIV/AIDS (PLWHA) in low- and middle-income countries were receiving ARV therapy [1]. In 2013, the World Health Organization (WHO) guidelines for HIV recommended systematically initiating treatment at an earlier threshold, a CD4 cell count ≤500. This recommendation looks forward to universal access to ARVs and controlling the spread of HIV transmission, although the number of eligible patients increased to 25.9 million and the need-coverage gap widened. The guidelines also recommended avoiding the use of stavudine for adult treatment because of its toxicity and to replace it with clinically superior tenofovir (TDF) in first-line regimens, or even substituting nevirapine by efavirenz (EFV) [2]. However, affordability remains a critical issue because regimens that include TDF or EFV are more expensive and impose extra pressure on funding agencies that are already confronted with budgetary constraints [3]. According to the UNAIDS, support from donor nations has reached a plateau since 2008, reflecting the economic and fiscal constraints of the post-financial-crisis period [4]. Low- and middle-income countries need an annual investment of US$24 billion to combat HIV by 2015, and at the current funding levels, there will remain a shortage of US$7 billion.

The question of affordability is crucial because an increasing number of patients need to switch to newer regimens because of side effects and treatment failure caused by treatment resistance. Indeed, the prices of newer first-, second- and third-line regimens are significantly higher compared with older first-line treatments because key ARVs are currently patented in developing countries. Competition between generic manufacturers (especially from India and Thailand) and multinational pharmaceutical companies was the main driver in bringing down ARV prices in the last decade [5]. Buttressed by their weak patent regimes, these countries could produce and export generic versions of patented medicines. However, since 2005, the enforcement of the World Trade Organization's (WTO) Agreement on Trade-Related Aspects of Intellectual Property Rights (TRIPS) in most developing countries requires them to grant pharmaceutical medicine patents for a period of 20 years and imposes additional restrictions on generic ARV supplies. In the present WTO framework, a patent on a single compound in a fixed-dose combination may block its production by generic manufacturers, leading to a monopoly in the market.

Shifting ARV demand, as countries choose less toxic but more expensive medicines, poses strategic challenges for international funding agencies that rely heavily on the supply of generic medicines to successfully run their HIV treatment access programmes. Understanding the ARV market and price determinants is, thus, essential to developing new policy measures to minimize ARV prices and implement more cost-effective ARV procurement. The aim of this study is to provide a landscape of the global ARV market by analyzing the transactional data on donor-funded ARV procurement between 2003 and 2015 and the ARV price determinants. We also examine the evolution of ARV prices through the initial patent life cycle, which might provide some insight into the pricing behaviour of pharmaceutical firms.

## Materials and methods

### Dataset

Our analysis is based on the effective prices paid for ARV procurement in low- and middle-income countries between 2003 and 2015. The data were obtained from the Global Price Reporting Mechanism (GPRM) managed by the AIDS Medicines and Diagnostics Service of the WHO, and it consists of information that covers approximately 80% of the total donor-funded ARV transactions provided by GPRM partners (Appendix 1). Information from the US Food and Medicine Administration (FDA) market approval dates was included as a proxy for the first launch in any market to calculate the length of medicines in the market; HIV/AIDS guidelines from the WHO allowed the construction of a four-category variable that classified medicines according to the therapeutic line in which they are recommended and that were time-varying according to the WHO guidelines [6]. Gross national income per capita (GNIpc) from the World Bank accounted for countries’ economic contexts and was imported to the dataset as a time-varying variable. Finally, the initial patent expiration year was obtained from the Medicines Patent Pool (www.medicinespatentpool.org/) to calculate the years before and after expiration at the time of purchase of both originator (i.e. medicines under patent protection when launched onto the market) and generic medicines (i.e. bioequivalent products). We assumed that the latter transactions were made under either voluntary or compulsory license or sold by taking advantage of TRIPS flexibilities.

Only adult ARVs were selected, resulting in a sample that comprised 64,423 transactions for 126 developing countries and that referred to 45 single formulations, 10 co-blister combinations and 27 fixed-dose combinations. ExWorks prices (no charge included) and procured quantities were standardized according to the guidelines in terms of yearly doses.

Number of yearly treatments per transaction per medicine

QYD=(number of smallest units)/[(units in daily dose)×(365 days)]

Price per yearly dose

PYD=(unit price in US$)×(units in daily dose)×(365 days)

### Multivariate approach

Ordinary least squares estimator was implemented, with time and geographic fixed-effects (pooled estimation). The natural logarithm of PYD was used for easier interpretation of the estimates in the following empirical model:

$$\log{\left(\text{P}YD\right)}_{it}=\alpha +\gamma \log(QYD)+\delta \log(GNIpc)+\beta X_{it}+\in_{it}$$

with *i=each transaction; t=year order*

The covariates, *X*, were the natural logarithm of both GNIpc and QYD; a three-category variable controlled for the type of formulation effect on prices (single, co-blister and co-formulations); length of medicines in the market, indicated whether five years or more had passed since FDA approval (=1) or not (=0); number of suppliers observed for each medicine per year; a four-category variable accounted for therapeutic lines (first-line, second-line, both first- and second-line and potential third-line) and a supplier type indicator (originator=1, generic=0). Fixed-effects dummy variables were specified for both the observed years of transaction (2003–2015) and World Bank developing regions: East Asia and Pacific (EA&P); Latin America and the Caribbean (LA&C); the Middle East and North Africa (MENA); South Asia (SA); sub-Saharan Africa (SSA) and Europe and Central Asia (E&CA).

We relaxed the unrealistic assumption of similar price structures in both segments imposed by the pooled estimation by a separate estimation for each type of supplier (originator/generic): the pertinence of the stratification was verified by an *F*-test (see Appendix 2). To depict the price patterns *vis-à-vis* the initial patent's life cycle, additional estimations were performed for each segment (originator/generic), including a variable indicating the number of years before and after initial patent expiration at the time of the transaction and its polynomial structure. For the generic segment, years before initial patent expiration refers to medicines that we assume to be sold either under license (both compulsory and voluntary) or sold by taking advantage of TRIPS flexibilities.

## Results

**Figure 1:**
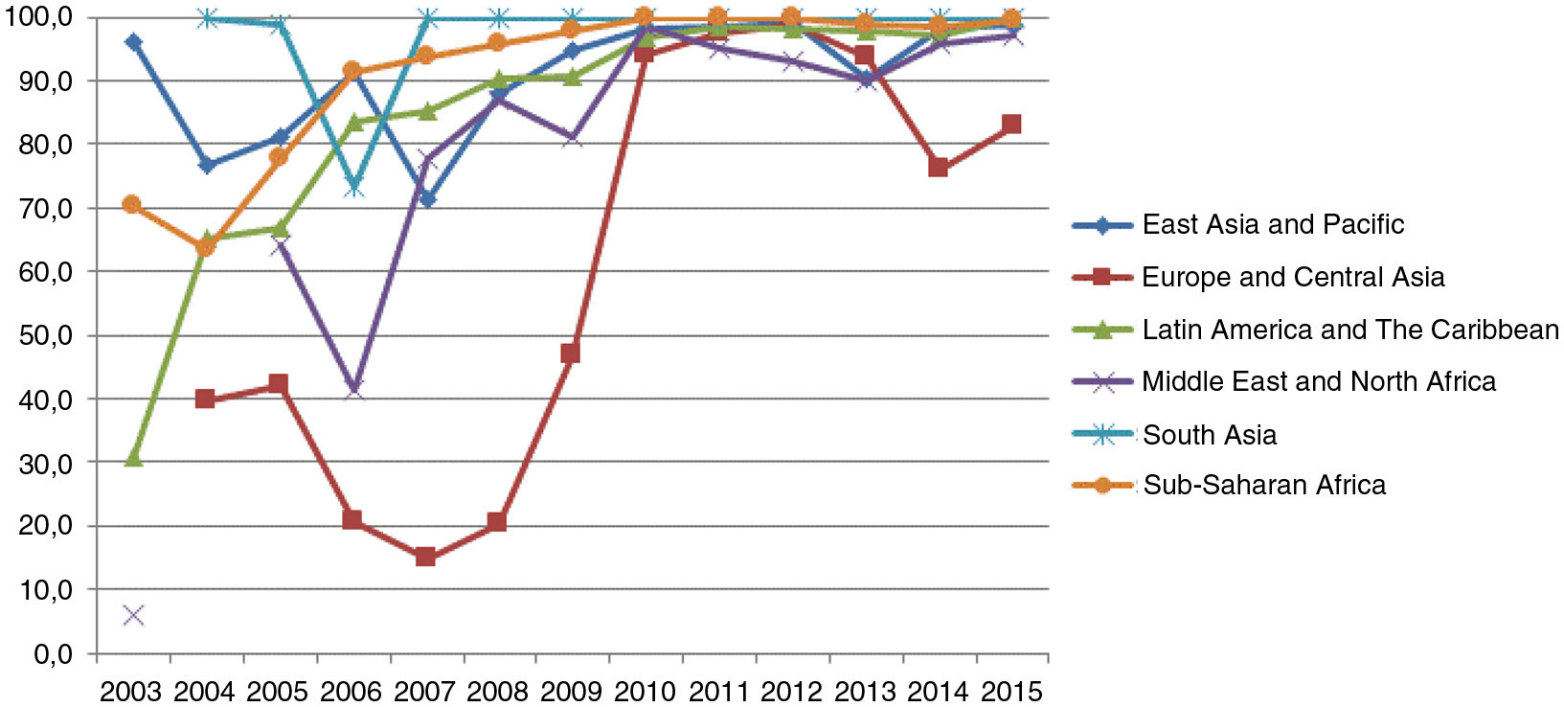
Proportion of procured generic yearly ARV treatments by geographic region (2013–2015).

The proportion of yearly generic ARV treatments increased from 71% of total purchases in 2003 to 97% in 2009 and remained stable around 99% between 2010 and 2015. Total expenditures for adult ARVs increased from US$63.3 million to US$1.53 billion between 2003 and 2015, with generics accounting for, respectively, 51.8 and 97.9%. Unsurprisingly, more than 94% of generic yearly treatments procured in this period were supplied by Indian manufacturers. Indeed, the market for generic ARVs for adults was controlled by a limited number of Indian firms during the observed period (i.e. Aurobindo, Cipla, Emcure, Hetero Medicines, Macleods, Matrix, Ranbaxy and Strides). Market share (in volume, Figure 1) of the generic segment appeared stable in SA and EA&P, whereas it progressed rapidly in SSA and MENA and especially in LA&C. By contrast, the generic segment share appeared unstable in E&CA, where the proportion of generic ARV procurement decreased after 2005 (from 40% to only 15%) and recovered after 2007.

**Figure 2:**
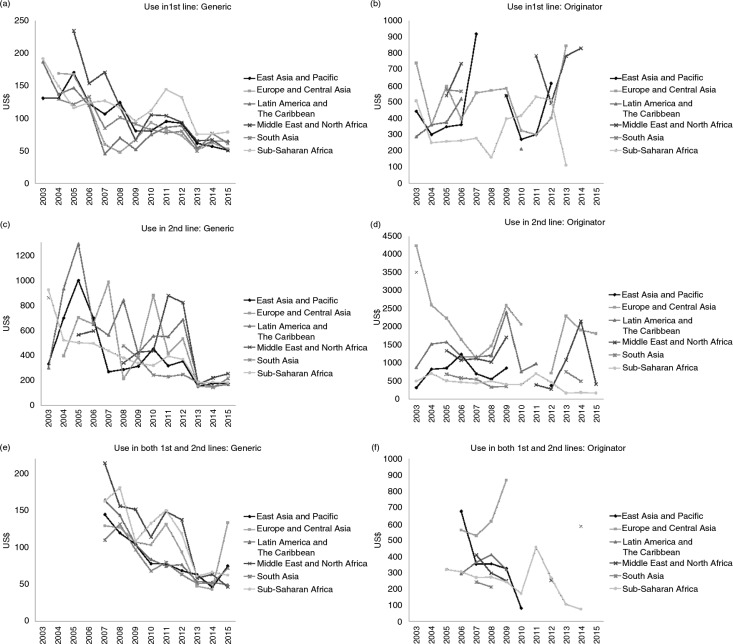
Evolution of the mean price of the adult treatment per patient-year by geographic region (2003–2015).

Adult ARV prices for yearly treatments decreased from US$464.3 on average in 2003 to less than US$136.8 in 2015. This reduction was driven by the decrease in generic prices (−69% by 2015 compared with the yearly treatment price of US$300 in 2003), whereas the average price of originator yearly treatments decreased by only 52% during the same period (with an average price of US$862 in 2003). Surprisingly, the lowest generic prices were not observed in the SSA countries where the HIV burden is the highest: in 2015, first-line generic ARVs were procured to SSA countries at an average price of US$78.3, whereas the costs were US$64.4 and US$50.2, respectively, for LA&C and E&CA countries (Figure 2a). In the same year, average generic prices for second-line medicines were similar (approximately US$179) in the SSA region but were lower in E&CA (US$170) (Figure 2c). By contrast, SSA countries paid on average the lowest originator prices (Figure 2b and 2d).

In order to explain the variations in prices observed in the preceding descriptive statistics, we estimated a multivariate model. Estimations for the general market (the “All” column in Table 1) show that originator medicines are on average 42.5% more expensive than generic medicines (*p*<0.001). However, price structure is not the same between segments (see Appendix 2). As shown by separate estimations in Table 1, originator medicines appear – on average – less expensive in the SSA and SA regions; prices for E&CA are on average 136% (*p*<0.001) higher than those paid in SSA countries. However, SSA seems to purchase generic medicines at the highest prices: for countries in both EA&P and E&CA, the price of generic medicines is respectively 24.4% (*p*<0.001) and 18.7% (*p*<0.001) lower than that for SSA countries. Another difference between the originator and generic segments is given by the effect of the countries’ economic conditions as measured by the GNIpc on prices. In fact, a 10% increase in a country's GNIpc is related to an increase of 1.01% in average generic prices (*p*<0.001).

**Table 1:** Price determinants in ARVs procured to developing countries (2003–2015)

	ALL (*n*=64,423)		Originator (*n*=11,312)^c^		Generic (*n*=53,111)^c^	
			
	Estimate	95% CI	Estimate	95% CI	Estimate	95% CI
Intercept	4.3867***	(4.3255, 4.4479)	5.3357***	(5.1576, 5.5138)	4.3662***	(4.3051, 4.4274)
Years						
2003	1.3447***	(1.1759, 1.5135)	0.6862***	(0.3230, 1.0495)	1.2851***	(1.1054, 1.4649)
2004	1.1078***	(1.0580, 1.1576)	0.8115***	(0.6920, 0.9309)	1.0207***	(0.9640, 1.0774)
2005	1.0167***	(0.9775, 1.0559)	0.8443***	(0.7410, 0.9475)	0.8746***	(0.8317, 0.9175)
2006	0.9120***	(0.8748, 0.9491)	0.7994***	(0.6979, 0.9009)	0.7919***	(0.7530, 0.8308)
2007	0.8835***	(0.8517, 0.9153)	0.7215***	(0.6276, 0.8155)	0.8482***	(0.8158, 0.8806)
2008	0.8550***	(0.8261, 0.8840)	0.7236***	(0.6364, 0.8109)	0.8637***	(0.8344, 0.8929)
2009	0.7355***	(0.7043, 0.7666)	0.6892***	(0.5839, 0.7945)	0.6788***	(0.6486, 0.7090)
2010	0.6588***	(0.6304, 0.6872)	0.7073***	(0.5866, 0.8281)	0.6459***	(0.6189, 0.6728)
2011	0.7461***	(0.7163, 0.7760)	0.6750***	(0.5580, 0.7920)	0.7369***	(0.7084, 0.7653)
2012	0.6885***	(0.6583, 0.7186)	0.4984***	(0.3893, 0.6075)	0.6861***	(0.6570, 0.7152)
2013	0.0416***	(0.0169, 0.0662)	0.2387***	(0.1586, 0.3189)	0.0186	(−0.0050, 0.0423)
2014	0.0277**	(0.0016, 0.0537)	0.2003***	(0.1203, 0.2803)	−0.0078	(−0.0330, 0.0174)
Ref: 2015						
Geographical regions^a^						
East Asia and Pacific	−0.1228***	(−0.1425, −0.1030)	0.5867***	(0.5216, 0.6518)	−0.2438***	(−0.2628, −0.2249)
Europe and Central Asia	0.1585***	(0.1339, 0.1832)	1.3595***	(1.3002, 1.4188)	−0.1873***	(−0.2126, −0.1621)
Latin America and the Caribbean	0.0338***	(0.0126, 0.0551)	0.9003***	(0.8437, 0.9569)	−0.1728***	(−0.1939, −0.1517)
Middle East and North Africa	0.1589***	(0.1165, 0.2014)	0.9781***	(0.8738, 1.0824)	−0.0858***	(−0.1288, −0.0428)
South Asia	−0.1407***	(−0.1767, −0.1047)	0.3606***	(0.2040, 0.5173)	−0.1865***	(−0.2201, −0.1529)
Ref: Sub-Saharan Africa						
log(Gross National Income per capita)	0.0768***	(0.0710, 0.0825)	−0.0275***	(−0.0432, −0.0117)	0.1012***	(0.0955, 0.1070)
log(Quantity purchased per transaction)	−0.0476***	(−0.0498, −0.0453)	−0.0481***	(−0.0554, −0.0409)	−0.0563***	(−0.0585, −0.0541)
Formulation type						
Co-blister	1.7479***	(1.6167, 1.8790)			1.8119***	(1.6939, 1.9300)
Co-formulation	0.4583***	(0.4457, 0.4708)	0.3680***	(0.3322, 0.4039)	0.5012***	(0.4888, 0.5137)
Ref: single formulation						
Drug age since FDA approval						
≥5 years	−0.2780***	(−0.3030, −0.2529)	0.2178***	(0.1507, 0.2848)	−0.3604***	(−0.3856, −0.3353)
Ref: <5 years						
Number of observed suppliers	0.0173***	(0.0160, 0.0186)	−0.0308***	(−0.0359, −0.0256)	0.0259***	(0.0247, 0.0271)
Therapeutic line^b^						
Used in 1st line	−0.9542***	(−0.9706, −0.9378)	−0.7005***	(−0.7525, −0.6485)	−1.0093***	(−1.0261, −0.9925)
Used in 3nd line	1.2245***	(1.1596, 1.2895)	1.1724***	(1.0900, 1.2549)		
Used in both 1st and 2nd line	−0.9510***	(−0.9671, −0.9348)	−0.5054***	(−0.5535, −0.4573)	−1.0509***	(−1.0676, −1.0342)
Ref: Used in 2nd line						
Market segment						
Originator	0.4251***	(0.4076, 0.4426)				
Ref: Generic						
Adjusted R2	0.509		0.410		0.477	
Sum of squared residuals	33,554		6525		22,349	

Significant at: *10%, **5% and ***1% confidence levels.

a World Bank classification of developing regions

b given that the dataset used in the study comprises transactions from 2003 to 2015, the therapeutic class accounts for the changes in WHO guidelines (time-varying variable)

c the F-statistic: 209.7 is higher than the critical value for the F-distribution with (24, 64, 371) degrees of freedom (approximately 1.57 at the 5% confidence level). The null hypothesis is rejected, indicating that separate estimations for the originator and generic segments better fit the data than the pooled estimation.

Both originator and generic prices are responsive to changes in the demanded quantities in a similar proportion: prices decrease, respectively, by an average of 0.48% (*p*<0.001) and 0.56% (*p*<0.008) with an increase of 10 in procured volume. Prices are sensitive to new suppliers of the same medicine: each additional supplier results in a decrease of 3.8% (*p*<0.001) in the originator segment, whereas in the generic segment an additional supplier results in an increaseof and 2.6% (*p*<0.001). In terms of length of the medicine in the market and therapeutic lines, our estimations offer the expected results.

**Figure 3:**
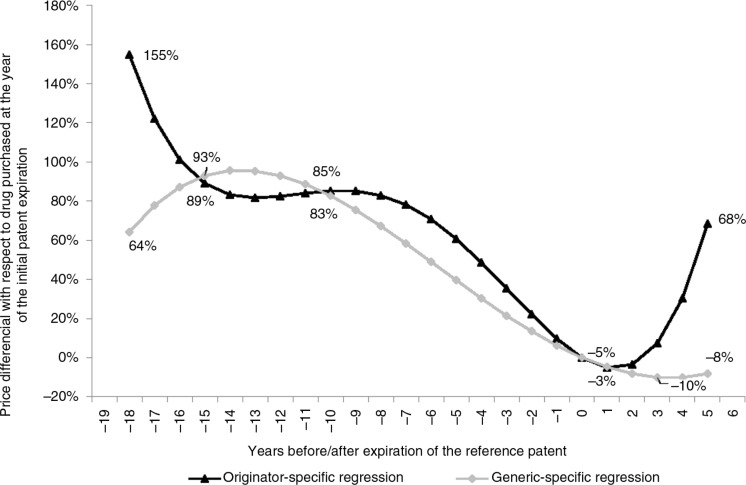
Evolution of originator and generic prices through the patent life cycle.

The evolution of price differentials through the patent life cycle was calculated using the polynomial estimates in Table 2. Originator medicines purchased 18 years before the expiration of the patent are on average 155% more expensive than medicines purchased in the year of patent expiration (the black line in Figure 3). Generic medicines (assuming that they were sold either licensed or taking advantage of TRIPS flexibilities) purchased 18 years before the expiration of the initial patent are on average 64% more expensive than generic medicines whose patent just expired. From the half-life of patents (10 years before expiration), the price patterns of both the originator and the generic segments become similar, although they evolve at different paces: one year after the expiration of the initial patent, originator and generic medicines are, respectively, 3 and 5% less expensive than they were in the year of the initial patent expiration. However, five years after the expiration of the initial patent, prices in the originator segment are 68% more expensive than they were in the year of the initial patent expiration; whereas in the generic segment prices are still less expensive (−8%), although increasing with respect to generic medicines at the year of the initial patent expiration.

**Table 2:** Polynomial specification of the years before/after expiration of the initial patent (2003–2015)

	Originator (*n*=11,312)		Generic (*n*=53,111)	
		
	Estimate	95% CI	Estimate	95% CI
Polynomial specification^a^				
Years before/after expiration of the reference patent	−0.0773***	(−0.0859, −0.0687)	−0.05541***	(−0.0580, −0.0528)
(Years before/after expiration of the reference patent)^2	0.0228***	(0.0211, 0.0245)	0.00643***	(0.0061, 0.0068)
(Years before/after expiration of the reference patent)^3	0.0034***	(0.0031, 0.0036)	0.00031***	(0.0003, 0.0004)
(Years before/after expiration of the reference patent)^4	0.00012***	(0.00011, 0.00013)	−0.000006***	(−0.00001, −0.000003)
**Controlling for the same effects as in Table 1**				
Adjusted *R*2	0.482		0.556	
Sum of the squared residuals	6525		18957	

Significant at: *10%, **5% and ***1% confidence levels.

a As suggested by the polynomial regression technique, exponents were introduced until the estimated coefficients were not significant.

## Discussion and conclusions

The vast literature on affordability and the scale-up of HIV/AIDS treatment contrasts with the scarcity of studies on price determinants using global data on medicine transactions [7–13]. To our knowledge, this study is the first to exploit the entire data on effective procurement prices in developing countries as collected by the new GPRM.

Existing literature on ARV price determinants does not reflect the global market because analyses are conducted using either transactional data at the regional level [10,12]; data provided by a reduced number of funding sources [8] or data from the old GPRM at the medicine level (old GPRM reporting not comparable prices in terms of the international commercial terms) [11]. Separate estimations better fit the data and are one of the main contributions of this article. This implies that originator and generic prices react differently not only to the strategies adopted by the manufacturers but also to other observable and/or unobservable factors (e.g. types of procurement, IP barriers or even policy aspects).

### Geographic price patterns differ between originator and generic ARVs

Originator medicines are on average the least expensive in the SSA region, whereas at the same time, generic medicines are on average the most expensive. By contrast, originator medicines are the most expensive in E&CA and generic medicines the least expensive. Given the extremely small part of the generic market in the E&CA region (scarcely 0.8% over the whole period vs. 88.8% in the SSA region), generic manufacturers appear to adopt low prices as a strategy of entry into the E&CA region at the same time that the huge amounts of generic ARVs procured to SSA (sold at the highest prices) would be compensating for the lower prices available in other markets. Sensitivity estimations were carried out by isolating the effect of South Africa in order to avoid a distortion introduced by patterns unique to this country (see Appendix 3). The results of the sensitivity analysis confirm the robustness of our results. In fact, our results suggest mixed strategies by ARV suppliers to exploit opportunities for profit maximization and to adapt to the specific conditions of market competition in each region. Price discrimination across regions could be reflecting a generic firm oligopoly that progressively drives the worldwide supply, maximizing profits by segmenting markets according to consumers’ willingness to pay [10]. This would not be so striking if we take into account the findings of Meiners *et al*. [12], who suggested that the Brazilian generic segment is not as competitive as it was thought. Transaction costs could be a second explanation for the generic price patterns across geographic regions, indicating the different payment capabilities of countries: payment delays could imply additional credit costs [14,15]. Unfortunately, information about payment terms (release of funds) and other aspects concerning negotiations remain unclear, and the variability of prices caused by these factors cannot be quantified. A third potential explanation for the different patterns of generic prices across the regions is related to the existence of different procurement types – “blind trust,” “systematic distrust,” and “cooperation” (i.e. over-the-counter transactions conducted directly by two parties in a less formal framework) – whose characteristics refer mainly to the purchasers’ power of negotiation and the way in which prices are fixed. Indeed, price differentials across regions could reflect the complexity introduced by adopting different procurement types, which are also dependent on the purchasers’ economic power. The “blind trust” strategy of negotiation adopted by the International Dispensary Association and the United Nations Children's Fund is consistent with the features of our dataset. These procurement agencies negotiate important proportions of both generic and originator medicines that are procured to the E&CA region (35.5 and 22.8%, respectively). Although the main advantage of this type of procurement lies in the very low transaction costs that could be the basis for the lowest generic prices estimated for the E&CA region, manufacturers may exploit the “passive” role of purchasers to set prices that will not be verified, which could be an alternative explanation for the highest originator prices estimated for E&CA. This result highlights the need for transparency in negotiation methods to improve the understanding of ARV market dynamics. Finally, the low quantities of generic ARVs procured to the E&CA region associated with the lowest prices could also reflect problems related to intellectual property (IP), which is the fourth (but not the least important) possible explanation for the pattern differences across geographic regions. Uncertainty about IP status created by the inefficiency (i.e. problems in recording and/or providing information about existing patents) of some offices is one of the main barriers that have been identified for the entry of generics in a given market. Consequences refer not only to impaired generic medicine procurement but also to the opportunity that uncertainty offers to procure more expensive patented medicines [16]. These features appear to effectively describe the estimations concerning the E&CA region (i.e. lowest generic prices but highest originator prices), where the difficulty in assessing the use of TRIPS flexibilities [16–18] underlines the lack of promotion for improving access to ARV therapy by introducing generic medicines (see, e.g. the Ukrainian situation, where the inefficient functioning of its IP office has been found to be one of the main barriers to the entry of generic medicines [19]). The lowest generic prices in E&CA could be reflecting the inefficiency of IP offices in the ARV market, where the only generic medicines procured are those whose IP status is certain. The impermeability of the ARV market in the E&CA region with regard to generic medicines could also suggest that this market is reserved not only for originator medicines but also for other generic medicines that were not reported on our dataset (i.e. local manufacturer medicines).

### Purchasing huge quantities of ARVs has modest impact on prices

Originator and generic prices are *inelastic* to changes in purchased quantities: prices were reduced by only 0.048% (*p*<0.001) and 0.056% (*p*<0.001), given 1% increase in quantities. This is in agreement with the WHO, WTO and WIPO (2011) discussions about the absence of price responses in the face of demanded quantities and the need to account for other aspects that intervene in the procurement process and that may have greater impact. In this sense, the voluntary pooled procurement service launched by the Global Fund in 2009 should pay attention not only to the incompatibility between negotiation terms and national regulations but also to the manufacturers’ willingness to participate in the negotiations. For the Andean region, these factors appeared to impair the effect of pooled procurement on prices [20]. Our results on the limited effect of purchased quantities on prices confirm the hypotheses of Lucchini *et al*. [10] and Meiners *et al*. [12] that monopsony (the presence of a single purchaser in a market) is able to compensate for monopoly power only when alternative suppliers are available.

### Prices show important variability through the patent life cycle

Using the year of registration of the initial patent in the European Union (EU) or in the United States as a *proxy* for ARVs’ IP status allowed for circumventing the lack of information at the country level [16]: the domination of the WTO system and its decision-making process by large markets, especially those of the EU and the United States in spite of the presence of 153 members [21], justify this choice. Strikingly, prices appear to be sensitive to the patent life cycle after controlling for other factors. The prices of generic ARVs that are produced under license or taking advantage of TRIPS flexibilities are not the same depending on whether the reference patent is at the beginning or the end of its life. This fluctuation in prices prior to the expiration of the initial patent could be explained by the royalties that generic manufacturers have to pay to their originator counterparts. The complex set of factors that determine these royalties (e.g. the therapeutic value and clinical superiority of the medicines, patients’ ability to pay, cumulative global revenues, and public health exigencies, among other factors), together with the scarce information about procedures for issuing a voluntary or compulsory license, contributes to the uncertainty in ARV prices [22]. Our findings show that generic ARV prices reach a minimum near the expiration of the initial patent and then increase again along with originator prices at a similar pace. This is contrary to the idea that the generic segment prices are close to the production costs and, by consequence, there is no more room to reduce prices [23].

In the medicine markets of developed countries, generic medicine usage and challenges to brand-name medicines’ patents have increased markedly since the 1980s [24]. In these markets, health economics research has highlighted the “generic competition paradox”: at the expiration of patents, generic entry is associated with fierce price competition, but this competition appears to be confined to the generic segment; patent owners generally do not follow price competition but rather increase their prices and attempt to protect their market share through product differentiation and marketing efforts [25]. Our results confirm that generic firms adopt similar behaviour following patent expiration in the ARV medicine markets of developing countries. Price differentials for generic medicines (that we assume sold either licensed or under TRIPS flexibilities) remain substantially high 10 years before the expiration of the initial patent, decrease rapidly until expiration and finally adopt a pattern similar to that of the originator prices, although at slower pace. This suggests that generic firms also tend to increase their prices after the initial patent expiration and may be attributable to the progressive move towards an oligopolistic structure in the generic segment controlled by a limited number of Indian firms. Indeed, in developed countries, generic medicine prices fall with an increasing number of competitors, and they remain above long-run marginal costs until there are a high number of competitors (eight or more in an estimation for the US market) [26]. Whereas increased patent protection has been associated with increases in R&D effort for new medicines in wealthy countries, the introduction of patents in developing countries has not been followed by greater R&D investment in the diseases that are most prevalent in those countries [27]. Our results suggest that the expiration of patents is not sufficient to boost additional developments in generic competition (at least in the ARV market) and that formal or informal agreements between generic firms may *de facto* slow down or even reverse long-term trends towards price decreases.

### Limitations of the analysis

Our dataset consisted of only donor-funded transactions and did not include ARV procurements by nationally funded HIV treatment programmes; thus, our results might not extend to national procurement settings. Another limitation of our study relates to the lack of information at the level of transactions regarding the terms of negotiation, release of funds and types of procurement including the mechanisms of large pooled procurement supported by the Global Fund and PEPFAR, all of which prohibited us from quantifying the variability in prices that was attributable to these factors.

## Concluding remarks

A market characterized by a demand shift towards clinically superior, but expensive medicines and strong IP regulations pose challenges to ARV therapy access in many developing countries. In the face of numerous constraints, additional price reductions for the recommended ARVs are essential for the long-term sustainability of HIV treatment access programmes. Our findings provide an improved understanding of the ARV market that can help countries strengthen policy measures to increase their bargaining power in price negotiations and the use of TRIPS flexibilities, with a special emphasis on negotiations with generic manufacturers.

## Appendix 1. Global price reporting mechanism partners that provide information about ARV transactions.

Detailed information about ARV transactions is provided to GPRM by major donors such as PEPFAR (President's Emergency Plan For AIDS Relief), CHAI (Clinton Health Access Initiative), Crown Agents, GDF (Global Medicine Facility), GFATM (The Global Fund to Fight Aids, Tuberculosis and Malaria), IDA (International Dispensary Association), Missionpharma, MSH (Management Sciences for Health), PFSCM (Partnership for Supply Chain Management), UNDP (United Nations Development Program), UNFPA (United Nations Population Fund), UNICEF (United Nations Children's Fund), UNITAID, USAID/Deliver and WHO/CPS (Contracting and Procurement Service).

## Appendix 2. *F*-test for validating the stratification of estimations by type of supplier (originator/generic).

The null hypothesis, in which the stratified model does not fit significantly better than the pooled model, is tested by using the following *F*-test:

$$F=\frac{\frac{RSS_{1}-RSS_{2}}{P_{2}-P_{1}}}{\frac{RSS_{2}}{n-P_{2}}}$$

where *RSS*_*i*_ and *P*_*i*_ are, respectively, the residual sum-of-squares of the model and the number of parameters of the model, with *I=1 (pooled), 2 (stratified)*; and *n* is the number of observations in the total sample. This test has an *F* distribution with (*P*_*2*_*-P*_*1*_*, n-P*_*2*_) degrees of freedom. Rejecting the null hypothesis would imply that prices are determined differently in both the originator and the generic segments of the ARV market (i.e. the price structures follow different patterns).

## Appendix 3. Price determinants in ARVs procured to developing countries (2003–2015): sensitivity analysis by removing South Africa from the Sub-Saharan Africa region.

**Table:**

	Originator (*n*=11,312)		Generic (*n*=53,111)	
		
	Estimate	95% CI	Estimate	95% CI
Intercept	4.1783***	(3.9669, 4.3898)	4.5369***	(4.4678, 4.6060)
Years				
2003	0.8568***	(0.4989, 1.2147)	1.3191***	(1.1394, 1.4988)
2004	0.8881***	(0.7703, 1.0059)	1.0281***	(0.9714, 1.0847)
2005	0.9185***	(0.8166, 1.0204)	0.8752***	(0.8324, 0.9181)
2006	0.8499***	(0.7499, 0.9500)	0.7928***	(0.7540, 0.8317)
2007	0.7373***	(0.6448, 0.8298)	0.8559***	(0.8235, 0.8884)
2008	0.7553***	(0.6694, 0.8413)	0.8706***	(0.8414, 0.8999)
2009	0.7141***	(0.6105, 0.8178)	0.6890***	(0.6587, 0.7193)
2010	0.7538***	(0.6349, 0.8727)	0.6501***	(0.6231, 0.6770)
2011	0.7272***	(0.6120, 0.8425)	0.7407***	(0.7123, 0.7691)
2012	0.5707***	(0.4631, 0.6783)	0.6863***	(0.6573, 0.7153)
2013	0.2088***	(0.1299, 0.2877)	0.0243**	(0.0007, 0.0480)
2014	0.1883***	(0.1096, 0.2671)	−0.0030	(−0.0281, 0.0222)
Ref: 2015				
Geographical regions^a^				
East Asia and Pacific	0.3170***	(0.2473, 0.3868)	−0.2070***	(−0.2272, −0.1869)
Europe and Central Asia	0.9347***	(0.8619, 1.0074)	−0.1289***	(−0.1565, −0.1014)
Latin America and the Caribbean	0.5855***	(0.5212, 0.6498)	−0.1162***	(−0.1399, −0.0925)
Middle East and North Africa	0.5887***	(0.4786, 0.6988)	−0.0303	(−0.0746, 0.0139)
South Asia	0.2387***	(0.0841, 0.3934)	−0.1618***	(−0.1957, −0.1279)
South Africa	−0.6794***	(−0.7488, −0.6099)	0.1267***	(0.1027, 0.1507)
Ref: Sub-Saharan Africa				
log(Gross National Income per capita)	0.1649***	(0.1399, 0.1900)	0.0698***	(0.0616, 0.0781)
log(Quantity purchased per transaction)	−0.0724***	(−0.0799, −0.0648)	−0.0523***	(−0.0547, −0.0500)
Formulation type				
Co-blister	0.3890***	(0.3536, 0.4244)	1.8476***	(1.7295, 1.9658)
Co-formulation			0.5050***	(0.4926, 0.5175)
Ref: single formulation				
Drug age since FDA approval				
≥5 years	0.2433***	(0.1773, 0.3093)	−0.3647***	(−0.3898, −0.3396)
Ref: <5 years				
Number of observed suppliers	−0.0266***	(−0.0316, −0.0215)	0.0262***	(0.0249, 0.0274)
Therapeutic line^b^				
Used in 1st line	−0.6712***	(−0.7225, −0.6200)	−1.0166***	(−1.0334, −0.9998)
Used in 3nd line	1.1431***	(1.0619, 1.2243)		
Used in both 1st and 2nd line	−0.4626***	(−0.5101, −0.4150)	−1.0579***	(−1.0746, −1.0411)
Ref: Used in 2nd line				
Adjusted *R*2	0.429		0.478	
Sum of squared residuals	7194		22,304	

a World Bank classification of developing regions

b given that the dataset used in the study comprises transactions from 2003 to 2nd quarter 2015, the therapeutic class accounts for the changes in WHO guidelines (time-varying variable)

c as suggested by the polynomial regression technique, exponents were introduced until the estimated coefficients are not significant. Significant at *10%, **5% and ***1%.
